# Supplementary material for: Are carbon emissions trading and green financial instruments synergistic? -Comprehensive quantitative research based on content analysis
Source: PLoS One. 2024 Mar 7;19(3):e0298601. doi: 10.1371/journal.pone.0298601 (PMC10919601; doi:10.1371/journal.pone.0298601)
Supplement: S1 Data — (ZIP) [file pone.0298601.s001.zip › Data/地级市Z_F工作报告.zip!/║ú─╧/╚2╤╟╩╨/└┤╫╘╣1⁄2╓┌║┼í╛┬φ┐╦╩2╛▌═°í┐_2018.docx]

各位代表:

现在,我代表市政府向大会报告工作,请予审议,并请市政协委员和其他列席人员提出意见。

一、2017年工作回顾

2017年是三亚升格为地级市30周年,也是新一届政府的开局之年。一年来,在省委、省政府和市委的坚强领导下,在市人大、市政协的鼎力支持下,市政府坚持以习近平新时代中国特色社会主义思想为指导,认真贯彻党的十八大、十八届历次全会和党的十九大精神,坚决落实省第七次党代会、省委七届二次、三次全会和市第七次党代会、市委七届历次全会的部署,围绕当好建设美好新海南排头兵的目标,启动实施“双十行动”,紧紧依靠全市人民,团结奋进,创新发展,完成了市七届人大二次会议确定的目标任务,实现了本届政府的良好开局。

(一)加快产业转型升级,经济发展稳中有进。2017年全市实现生产总值529.3亿元,增长7.6%;固定资产投资868.1亿元,增长10.9%;社会消费品零售总额223.8亿元,增长13%;地方一般公共预算收入93亿元,增长10.1%。三次产业结构为12.6:20:67.4,第三产业的比重比2016年提高了1.3个百分点。对标我省十二个重点产业,按照“一支柱两支撑”的产业格局,谋划培育十大产业,旅游、热带特色高效农业、新兴科技、医疗健康、会展、金融、物流、教育文化体育等重点产业发展较快,全年增加值增长9.6%,增速比整体经济高2个百分点。着力丰富旅游产品,南山“4+2”等项目加快推进,“三亚—菲律宾”邮轮航线开通运营,太阳马戏演艺节目正式上演,亚特兰蒂斯、红色娘子军演艺、启迪冰雪运动中心即将开业,海棠湾水稻国家公园、中廖村、文门村等美丽乡村广迎宾客,开通了3条“大三亚”旅游经济圈城际公交线路,区域协调合作、产品开发、市场推介取得初步成效。继续完善“四位一体”旅游市场监管机制,加强常态化管理,上线运行“三亚放心游”APP,切实维护游客合法权益。全市接待过夜游客1831万人次,增长10.9%,其中入境游客69.3万人次,增长54.4%,创历史新高;旅游总收入406.2亿元,增长26%,其中旅游外汇收入5.3亿美元,增长108.3%。金融业增加值63.7亿元,占全市生产总值的12%。深海科技产业园正抓紧谋划,启迪之星、海南软件创新中心的孵化企业落户三亚,市高新技术产业园的互联网信息产业产值增长了两倍多。医疗健康产业方兴未艾,三亚成为全国首批健康旅游示范基地之一。

(二)推进“五网”基础设施建设,城市功能逐步完善。凤凰机场三期项目加快推进,机场旅客吞吐量达到1939万人次,增长11.6%。三亚国际邮轮港二期项目稳步实施。有轨电车示范线首通段已开展联调联试。三横路凤凰机场段等道路通车,三亚大桥重建、海榆东线海棠湾至亚龙湾段道路改扩建工程即将实现功能性通车,亚龙湾第二通道等项目扎实推进。加强变电站建设,完善城乡电网线路,农村电网薄弱、电压质量不高等问题得到有效缓解。扎实推进西部供水、大隆灌区配套工程、田洋灌排渠道整治等水利工程,推进青田水厂、海棠湾水厂等水厂扩容,日供水能力提升到61万吨。天然气城市普及率达100%。城区小区和行政村光网覆盖率达100%,三亚大数据中心正式启用。

(三)加强生态文明建设,环境优势巩固提升。坚决落实中央环保督察、国家海洋督察的要求,立行立改,拆除了洲际度假酒店“海上餐厅”、小洲岛度假酒店等违法建筑,持续开展生态环境六大专项整治,打造“双修”升级版。全面推行“河长制”,开展城镇内河(湖)水环境治理,实施3宗河道整治工程、2宗水系连通工程,启动全市农村区域生活污水综合整治,新建或扩建4座污水处理厂,新增13座移动式污水处理站,对违法排污行为进行严管重罚,消除了8条水体黑臭现象,18个监测断面水质达标率有所提升。有序开展19个受损山体修复,继续实施“绿化宝岛”行动,完成植树造林1501亩,依法退果还林1129亩,补植红树林189亩。扎实推进海岸带修复,加强大气、土壤污染防治。金鸡岭桥头公园、鹿回头滨河公园已完工,收回名花公园等7个公园绿地免费向市民开放,凤凰路等11条道路的绿化彩化已完成。地下综合管廊建设、中心城区海绵化改造、夜景灯光改造升级等项目有序推进。大力推广绿色能源,全市新注册登记新能源汽车2178辆,新增充电桩510个,市政府与法国电力集团签署了《三亚低碳智慧能源综合利用海棠湾示范区项目合作声明》。全年拆除违法建筑211.3万平方米,建筑废弃物处理厂建成试运行。完成了41个“无违建”社区(行政村)创建工作。严格落实“巩卫”责任,深入开展城乡环境卫生综合整治,全面完成31家农贸市场升级改造,完善环卫设施建设,强化餐厨垃圾联合执法,有力推进“厕所革命”,市容市貌显著改善,再次蝉联国家卫生城市。

(四)加快改革开放步伐,发展活力有效释放。成立三亚市规划委员会,在全省率先建立“多规合一”联审申报系统。深化行政审批制度改革,推行极简微申报、全流程互联网“不见面审批”,开通项目“绿色审批通道”,项目审批事项平均提速77%,2017年纳入“项目投资百日会战”的124个新开工项目全部实现开工。积极推行“多证合一”,全市新增市场主体2.45万户,增长数量比2016年多86.7%。有序推进国企重组、上市、托管、关停退出等工作,推进国有资本投资公司、运营公司改革试点,鹿回头景区委托管理工作已正式启动。推进强区扩权,实施市与区财政分税制管理,将棚户区改造征收、“十镇百村”规划建设、项目投资审批等事项下放到区。积极探索区直管村(居)体制,创新大数据、信息化技术应用,天涯区智慧城市试点建设有序推进,还被评为全国农村社区治理实验区。深入开展农垦改革,已设置了5个农场居,南新农场公司制改革稳步推进。成功举办第67届世界小姐总决赛、第47届世界青年帆船锦标赛、第12届中国邮轮产业发展大会暨国际邮轮博览会、第8届海天盛筵、首届世界瑜伽交流大会、首届三亚国际能源论坛、首届国际水稻论坛等赛事与活动。新开通至印度德里、乌克兰基辅等9条国际航空航线。南海佛学院一期建成招生,为开展佛教文化对外交流提供了平台。

(五)加大富民惠民力度,民生福祉持续增进。2017年全市民生支出占地方一般公共预算支出的77.1%。完成年度脱贫任务,全市共有77户272人脱贫退出,年度计划减贫人口和已脱贫人口的教育、医疗、住房“三保障”和产业帮扶实现全覆盖。城镇新增就业岗位3.4万个,农村劳动力转移就业1.27万人,城镇登记失业率1.54%。城镇常住居民人均可支配收入33638元,农村常住居民人均可支配收入14570元,分别增长8.2%和9.1%。7所公办幼儿园建成开园。引进西南大学全面托管市第六中学、海南中学全面托管市实验中学。人大附中三亚学校已开建,上海外国语大学三亚附中已落户。市人民医院急诊综合楼建成投用,市妇幼保健院新院区主体结构封顶,三亚恒大妇产医院、三亚国康医院等项目稳步推进,组建了4个医疗服务联合体,推进分级诊疗工作。市群众艺术馆建成投用,市文化中心、市体育中心规划建设已经启动,群众性文体活动和全民健身活动广泛开展。海坡、东岸片区棚改完成征拆,南边海环河口、阳光海岸、西瓜村、芒果村等片区棚改旧改加快推进,全年完成棚改任务8152户。国际旅游岛人才创业基地首期4539套公租房陆续交付使用。启动了11577套保障性住房建设。“菜篮子”工作取得新成效,全市平价蔬菜销售网点已超过70个。2017年居民消费价格比上年上涨2.9%,低于预期调控目标。试点施行“路长制”,坚持“一警多能、多警联动、齐抓共管”,加强了道路交通管理和社会治安综合治理,大力开展禁毒三年大会战行动,严厉打击违法犯罪活动,再次获评“全国社会治安综合治理优秀市”,第三次荣获“长安杯”。做好信访工作,认真倾听、依法解决群众合理诉求。积极支持国防建设,国防动员、人民防空、民兵预备役和双拥共建取得新成效,安全生产、民族宗教、外事侨务、人口计生、防灾减灾、食品药品安全、口岸、气象、统计、档案、史志等工作取得新成绩,妇女、儿童、青少年、老龄、残疾人、工会、慈善事业实现新发展。

(六)强化政府自身建设,工作效能不断提升。一年来,我们始终坚持旗帜鲜明讲政治,牢固树立“四个意识”,深入学习贯彻党的十九大精神,自觉用习近平新时代中国特色社会主义思想武装头脑、指导实践、推动工作,坚决落实上级各项决策部署。推进“两学一做”学习教育常态化制度化,抓好政府作风建设,加强督查督办,加强审计监督,加强廉政建设,提升了工作效率,查处了一批违法违纪案件。认真执行市人大及其常委会的决议、决定,自觉接受人大、政协监督,2017年办复市人大代表建议124件、市政协委员提案177件,办复率100%。

各位代表,过去一年的成绩来之不易,这得益于习近平新时代中国特色社会主义思想的指导,得益于省委、省政府和市委的坚强领导,是全市人民团结奋斗的结果。在此,我谨代表市政府,向全市各族人民,向人大代表、政协委员,向各民主党派、工商联、无党派人士,向各人民团体和社会各界人士,向中央和省驻三亚各单位,向驻市部队、武警官兵、公安政法干警,向所有关心支持三亚发展的国内外朋友,表示衷心的感谢!

在总结成绩的同时,我们也清醒地看到,我市发展不平衡不充分的问题还很突出,特别是产业结构不合理,房地产业占比较大,现代服务业发展水平较低,调整产业结构、提升发展质量的任务艰巨;对生态环保工作的认识还不到位,围填海项目审批不规范,执法监管不到位等问题比较突出,局部生态环境质量不容乐观;国际化水平不高,城市功能和治理管理亟待完善,与世界级滨海旅游城市的定位还有很大差距;教育、医疗等社会事业发展滞后,公共服务水平有待提高;一些政府工作人员的担当精神、创新意识、发展能力不足,“四风”问题依然不同程度地存在。我们一定正视问题,切实推进解决。

二、2018年重点工作

2018年是贯彻党的十九大精神的开局之年,是决胜全面建成小康社会、实施“十三五”规划承上启下的关键一年,是改革开放40周年和海南建省办经济特区30周年,也是习近平总书记视察海南发表重要讲话5周年,做好今年工作意义重大。政府工作的总体要求是:以习近平新时代中国特色社会主义思想为指导,全面贯彻党的十九大、中央经济工作会议和省委经济工作会议、全省“两会”精神,落实市委七届六次全会的部署,统筹推进“五位一体”总体布局和协调推进“四个全面”战略布局,坚持稳中求进工作总基调,践行新发展理念,推动高质量发展,以供给侧结构性改革为主线,以“双十行动”为抓手,加快产业转型升级,加强生态文明建设,不断深化改革开放,推动城乡统筹发展,补齐民生事业短板,加快建设世界级滨海旅游城市,努力当好建设美好新海南的排头兵。2018年的主要预期目标是:地区生产总值增长7%;固定资产投资、社会消费品零售总额、地方一般公共预算收入各增长10%;城镇和农村常住居民人均可支配收入各增长8%;居民消费价格涨幅控制在3.5%以内。重点抓好以下工作:

(一)推进供给侧结构性改革,加快构建现代化经济体系。围绕转型升级、提质增效的目标,加快产业结构调整,着力构建以大旅游为支柱、以新兴科技产业和热带特色高效农业为支撑的产业格局。

一是统筹发展十个重点产业。做精做强旅游业。扎实推进全域旅游示范市建设,当好全省全域旅游和旅游新业态发展的排头兵。加快南山“4+2”项目建设,继续推进天涯海角、亚龙湾热带天堂森林公园、西岛旅游区创建5A级景区,发挥亚特兰蒂斯项目的引领作用,加快海昌梦幻不夜城建设,继续引进世界级主题公园,规划建设专业化旅游商品市场和特色风情商业街。积极推动邮轮、游艇、婚庆、亲子旅游等业态发展,争取今年进出港邮轮达到170航次,出入港游客超过23万人次,游艇产业收入达到2.5亿元,婚庆产业总收入达到110亿元,亲子游总收入超过100亿元。结合海洋、遥感、南繁等科技发展,推进创意产业园鲸世界等项目,打造科技游产品。加快槟榔河大型实景演艺公园、崖城历史文化名镇建设发展,打造文化游产品。积极推动“大三亚”旅游经济圈基础设施互联互通,共同开发旅游产品和精品线路。加快培育新兴科技产业。当好全省发展新兴科技产业的排头兵。依托中船重工、中船工业、中科院深海所等“国家队”力量,加快推动三亚深海科技城建设,培育深海技术国家实验室。积极打造中国“南繁硅谷”,加快国家南繁基地三亚保护区和南繁育种科技城建设。大力发展互联网产业,建成市高新技术产业园互联网信息产业基地,加快吉阳互联网信息“双创”产业园、海棠湾IT产业园建设,推动启迪南方产业基地、京东智谷、东华软件智慧三亚等项目尽快落地,确保海南信息安全基地如期建成运营。加快建设遥感产业园,实施海南卫星星座项目,搭建高分遥感大数据云服务平台。大力发展热带特色高效农业。优化农业结构,提升农产品品质,培育新型经营主体,推行现代化生产方式,实施品牌化战略,提升农业发展质量和效益。积极发展医疗健康产业。加快推进国家健康旅游示范基地、中医药健康旅游示范区和国家医养结合试点建设,推进北大口腔医院三亚分院、三亚恒大妇产医院、石药德中健康产业园等项目,确保三亚国康医院建成开诊。加快发展金融业。丰富金融业态,鼓励分行业设立产业基金,积极推进亚太金融(基金)小镇、国际基金小镇建设发展。继续壮大会展业。按照“一个主要湾区一个会展中心”的布局,高标准推进三亚国际会展中心、三亚国际文化会展中心等项目建设。坚持“以会议带展览,以展览促产业”,结合我市产业发展方向,培育海天盛筵、文博会、婚庆节、芒果节等自主会展品牌,同时吸引国际知名会展进驻。着力发展现代物流业。积极推进梅村综合物流产业园建设、招商,加快完善物流基础设施和网络体系。坚决推动房地产业优化调整。坚持房子是用来住的、不是用来炒的定位,抓好房地产市场调控和监管、整治,严控普通商品住宅、普通度假酒店供地,引导房地产业与其他产业融合发展。特别是要结合棚户区改造,鼓励发展城市综合体,引进优质产业项目,发展金融、商务、商业等地产。加快发展教育文化体育产业。加快推进人大附中三亚学校、上海外国语大学三亚附中等项目,积极推动北大附中三亚学校落户。加快规划建设市文化中心、市体育中心、三亚大剧院等设施,办好2018年央视春晚三亚分会场、首届中国体育庙会、太阳马戏演出季、克利伯环球帆船赛、第二届世界瑜伽交流大会、首届三亚国际音乐节、国际舞蹈节、国际青少年合唱节、国际时装周等活动,确保每个月至少举办一项具有重大影响的赛事活动。积极发展海洋产业。在保护好生态环境的前提下,挖掘海洋资源潜力,向海洋要质量、要效益、要增长。大力推进三亚崖州湾海南热带海洋学院海洋牧场教学科研示范基地等项目建设,通过培育邮轮游艇、水上运动等业态,丰富海洋旅游内涵,全力支持深海科技产业发展,推动海洋渔业转型升级,加快提升海洋运输物流和服务保障能力。

二是扩大有效投资。努力推动投资规模与质量“双提升”。全市安排固定资产投资项目441个,年度计划投资954.9亿元,其中十大产业项目194个,年度计划投资557.5亿元。进一步完善项目管理机制,成立市级专项领导小组,组织精干力量做实做强领导小组办公室,强化市级领导督查,落实区级主体责任,加快项目建设进度,缩短资本形成周期。突出抓好产业投资,坚持用最好的资源吸引最好的投资者,明确各类产业项目的具体准入标准,坚决不上污染环境、破坏生态的项目,引入低能耗、低排放、高效益、高科技、绿色化的项目。加强产业园区规划建设,完善配套设施和管理体制,充分发挥集聚平台作用。开展精准招商,主动对接境内外500强、行业龙头企业,引进重大产业项目。进驻项目需签订产业用地发展协议书,各区和市有关部门要加强跟踪服务、跟踪监督,采取对赌、分期等方式供地,提高项目落地率、履约率,推动招商项目从“数量型”向“质量型”发展。

三是打好防范化解重大风险攻坚战。认真开展政府存量债务化解工作,保障按时还本付息。规范政府举债、融资行为,规范PPP合作模式和政府购买服务方式,坚决防止违规、变相举债。建立健全政府债务风险预警与应急机制,将债务率控制在合理范围内。加强金融风险动态监测、预警评估,拓展部门监管合作范围,完善金融安全防线和风险应急处置机制,优化区域金融生态环境。坚决打击房地产投机炒作,规范市场秩序,调整产品结构,促进房地产业平稳健康发展,逐步降低对房地产业的依赖。

(二)加强城市建设和治理管理,着力提升功能与品质。坚持高水平规划设计,高标准建设管理,打造功能完善、文明有序、宜居宜业的精品城市。

一是加快“五网”基础设施建设。大力推进三横路、亚龙湾第二通道等项目,推进农村公路六大工程,启动建设三亚绕城高速凤凰机场互通及连接线、亚龙湾互通及连接线改造等项目,开展环岛高速大三亚段改线等项目前期工作。依法依规推进新机场项目前期工作。加快完善三亚国际邮轮港二期工程,着重加强功能性开发与拓展。研究构建新机场及邮轮港综合交通体系。加快有轨电车示范线建设,确保尽快完工。积极推进海榆东线南段和胜利路地下综合管廊建设,建立健全管廊、管箱、管槽的投资和运营管理机制。着力提升电网可转供水平和供电可靠率,全市年户均停电时间要低于18小时,中心城区要低于4小时。扎实推进西水中调、岭曲引水、北部山区安全饮水工程,加快推进青田水厂远水工程扩建,保持后备供水能力,尽快实现我市农村饮水安全全覆盖。对全市农田灌溉和排洪排涝问题进行梳理整治,提高灌溉水利用系数,解决田洋排灌、排涝问题。加快推进育才、南丁等片区天然气管道建设,积极推动燃气下乡进村。动工建设第二气源站,增强气源保障能力。推动光网和4G网络向全部自然村覆盖,对城区原有4G基站进行扩容,提升信号质量。

二是实施棚户区改造工程。扎实推进南边海环河口、阳光海岸、西瓜村、芒果村、月川村等片区棚改旧改,加快建设棚改安置区,确保年内完成5014户棚改任务。科学规划设计,统筹利用好棚改片区的土地资源,合理布局公共服务和生活配套设施,引入优质产业项目,改善居住环境,提升城市品质。按照国际水准规划建设“凤凰海岸”项目。加强棚改居民就业培训和创业引导,让群众长期生活有保障。

三是开展城市“五化”工作。加强城市净化、绿化、彩化、亮化、美化。重拳打击违法用地、违法建筑,全面完成存量违建整治任务,全面开展“无违建”示范点创建,坚持抓早抓小,严控新增违建。全面清理不符合标准的户外广告,严格规范审批管理。深化“巩卫”成果,常态化开展城乡环境综合整治,实现中心城区保洁24小时全覆盖,加强城中村、背街小巷等薄弱环节卫生管理,落实“门前三包”责任,巩固“厕所革命”成绩,创新“三无弃管”小区管理模式。加快推进生活垃圾渗滤液处理站二期、焚烧发电厂三期和垃圾转运站项目,试点开展生活垃圾分类,推进建筑垃圾资源化利用,着力提高餐厨垃圾收集处理率、农村生活垃圾收运和无害化处理率。加快推进新岸湿地公园、抱坡溪湿地公园、荔枝沟社区公园、月川滨河公园、棕榈滩滨河公园、海坡中央公园、小洲岛公园等公园建设,完善城市绿道系统,对全市生态树池进行全面改造。加强公园、绿道维护管理,优选植物种类,优化种植布局,配建群众性体育设施,增强植物观赏和体育健身功能。继续实施机场路和海虹路等道路绿化彩化工程,完善电表箱、垃圾箱、书报亭、公厕等细节的彩化美化工作,积极推进居民阳台、屋顶、庭院等立体绿化,逐步实现城市主干道路两侧“阳台必须有绿”。加快实施城市夜景灯光改造升级工程。继续实施临春河两侧建筑风貌提升示范段、商品街大道商铺外立面改造、大东海片区花街改造和南边海渔村改造提升等工程,着力打造精品街道、精品社区。以“多规合一”为引领,积极开展城市设计工作,加强规划审批和后续监管,确保规划设计要求落实到位,逐步形成具有三亚特色的城市风貌。

四是提升治理管理水平。加强网格化、精细化管理,实施“片长+网格长+12345热线”管理体制,实行部门网格联动及限时办结机制;加强智能化、信息化管理,结合“智慧城市”建设,加快大数据的梳理、收集、分析、应用,推动各级、各部门数据共享和业务协同。结合创建全国文明城市工作,聚焦旅游市场、交通、治安等重点环节,创新治理管理措施。继续发挥“四位一体”旅游市场监管机制的作用,完善有奖举报办法,高效处理涉旅投诉,坚决实行严管重罚,大力推行诚信经营,强化主动性常态化执法检查;严格落实农贸市场、水果店、餐饮服务行业、旅游购物点等管理办法,按照全域旅游的标准,打造一批旅游购物、餐饮示范点;加快全市旅游监管与服务平台建设,提高旅游大数据的收集、应用水平;整合全市热线资源,完善服务热线平台功能。优化道路网络,加快机非分离、人车分离,加强停车场建设、停车位施划,科学疏导车流、人流,逐个片区、逐条街道彻底整治违停车辆,严查交通违法行为,规范交通秩序。推进综治中心、雪亮工程、网格化管理“三位一体”新机制建设,进一步完善“路长制”,推动警力下沉、警务前移,提高见警率和管事率。全力推进社会文明大行动,积极争创全国文明城市。强化社会治安综合治理,深入开展扫黑除恶专项斗争,持续开展禁毒三年大会战,严厉打击各类违法犯罪活动,打造平安三亚。进一步畅通信访渠道,落实信访工作责任制。抓好安全生产工作,预防和遏制重特大安全事故发生。加强灾害预警和应急救援体系建设。大力支持国防建设,抓好国防动员、民兵预备役和人防工作,广泛开展双拥共建活动。继续做好民族宗教、外事侨务等工作,支持妇女、儿童、青少年、老龄、残疾人、工会、慈善等事业发展。

(三)实施乡村振兴战略,做好新时代的“三农”工作。按照产业兴旺、生态宜居、乡风文明、治理有效、生活富裕的总要求,完善城乡融合发展体制机制,加快推进农业农村现代化,大力促进农民增收,当好区域协调发展和乡村振兴的排头兵。

一是高水平推进“十镇百村”建设。统筹抓好“十镇百村”建设与产业发展、全域旅游、脱贫增收、生态文明建设等工作,完善农村基础设施、公共服务,加快农村产业发展,加强农村环境整治,确保农民增收获益。积极推进亚龙湾玫瑰风情小镇、海棠湾潜水及水上运动特色小镇、青田中医康养·金融小镇、文化艺术小镇、光伏小镇等项目,抓好小镇招商和业态培育,结合演艺、电商、时装等业态发展,谋划新的特色小镇项目。坚持科学选点、科学规划,立足当地资源,合理改造提升,保持田园风光,突出特色风貌,加强产业培育,扎实推进博后、大茅、南山、藤海、抱前、文门、西岛渔村等美丽乡村示范村建设发展,新启动31个自然村的美丽乡村建设。着力实施区域协调发展战略,强化对育才、高峰等地区发展的引导和支持。

二是大力发展热带特色高效农业。深入推进农业供给侧结构性改革,继续抓好冬季瓜菜、热带水果、南繁育种、花卉苗木等产业,推进畜禽标准化、规模化养殖,优化种植、养殖结构,扩大优质高效农产品供给。加快渔业转型升级,继续推进骨干捕捞船队建设,压缩近海捕捞,发展外海捕捞,逐步取缔沿岸海水养殖,培育发展休闲渔业。稳步推进承包地“三权分置”和经营权流转改革,发展适度规模经营,打造新型农业经营主体,着力引进、培育国家级和省级龙头企业、农民专业合作社。推行农业标准化生产和全程质量控制,加强农产品品牌宣传和市场营销,做优做强“三亚芒果”“三亚莲雾”等品牌,新打造“三亚豇豆”等品牌。拓展完善岛外农产品销售网络,大力发展“互联网+农业”,“互联网+乡村综合体”,加强产销数据对接,创新开展“以销定产”的订单式生产。加快崖州果蔬综合批发市场二期等项目建设,积极扶持水果岛等龙头企业,建设一批精品连锁体验店。依托崖州中心渔港,打造海南南部渔业交易中心。加快农业发展“接二连三”,着力培育优势农产品精深加工,结合美丽乡村建设,开发田园综合体、果园综合体,争取今年新建5家共享农庄试点。

三是打好精准脱贫攻坚战。进一步完善扶贫大数据,注重扶贫与扶志、扶智相结合,着力发展可持续增收的产业,加强技能培训和产业帮扶,巩固脱贫成果。建立扶贫工作长效机制,完善教育、医疗、住房“三保障”等政策体系。今年要完成现有的28户贫困户共119人脱贫退出,并对2014年以来脱贫退出的2241户9951人进行分类巩固。扎实开展脱贫攻坚作风建设年活动,加强扶贫领域作风问题专项治理。

四是实施好新一轮农民增收计划。在打好精准脱贫攻坚战的基础上,千方百计促进农民增收。加强政策引导、技术指导,鼓励农民调优种植、养殖结构,提升农业生产的标准化水平。加强农业科技服务,开展农村实用人才培训,培养懂科学、有技术、会经营的新型职业农民。借助互联网和大数据,及时有效掌握农户生产和市场需求情况,充分利用游客消费市场,结合“菜篮子”工作,推动优质农产品上餐桌、入超市、进酒店,提升产品价值和农户收益。加强“农旅融合”,鼓励发展休闲农业。稳步推进农村集体产权制度改革,探索用好农民闲置宅基地和闲置农房政策,通过发展特色民宿等方式,增加农民财产性收入,同时加强监管,严禁在农村变相搞房地产开发,严禁城市居民到农村购买宅基地建房。加强就业服务,落实创新创业扶持政策,统筹抓好城镇新增劳动力和农村转移劳动力就业。结合旅游等行业发展和企业岗位需求,开展定向式、定岗式技能培训。加强农民小额贷款贴息和农业保险工作,确保各项惠农资金及时落实到位。结合农村基础设施建设,积极发展分布式光伏产业。

(四)坚持绿色发展,打造全国生态文明建设示范市。牢固树立“绿水青山就是金山银山”的理念,坚决贯彻落实市委“生态文明建设100条”,当好全省环境保护和生态文明建设的排头兵。

一是打好污染防治攻坚战。坚决贯彻落实中央和省委的要求,对照中央环保督察、国家海洋督察的反馈意见,立行立改,举一反三,抓紧整改各项突出问题,继续开展生态环境专项整治,加快“双修”升级版建设。实施大气污染防治行动,强化对秸秆焚烧、建筑工地、道路扬尘、黄标车污染、餐饮业油烟等方面的监管,实现全市无秸秆焚烧火点的目标,逐步推进在建建筑物金属网围挡降尘,确保三亚空气质量继续保持全国领先。加强水环境综合治理,加快推进红沙污水处理厂二厂、荔枝沟水质净化厂二厂、亚龙湾污水处理厂二厂建设,实施新城水质净化厂提标改造,启动吉阳人才城水质净化厂前期工作,扩建污水管网。加快海坡内河水系连通、高园水库与大茅水连通等工程,增强全市水资源统一调配能力和防洪减灾能力。完善和落实“河长制”“湾长制”“湖长制”“库长制”,扎实推进11条内河水环境治理、全市农村区域生活污水综合整治,完成18个监测断面水质管理目标,全市主要河流湖库水质优良率不低于95%。加强海岸带和海洋生态环境保护,开展珊瑚礁修复和保护蓝色海湾整治行动,实施铁炉港红树林保护区生态修复工程,启动三亚湾东段拦沙堤、三亚湾西段生态护岸、亚龙湾西段人工补沙与生态护岸等工程,恢复和提升海岸线功能。严查污水排海行为,制定防止船只污染的管理办法,加强崖州中心渔港入港渔船规范管理和环保监管。认真落实海洋环境评价制度,加强海岸带生态红线管控、填海用海动态管理以及建设工程审批监管。完成19个受损山体修复后续工作,新开工3个受损山体修复项目。继续开展“绿化宝岛”行动,依法实施退果还林工程。加快推进全国殡葬综合改革试点工作,治理乱埋乱葬、毁林建墓等行为。加强土壤环境综合治理,通过实施土壤地力提升和化肥、农药减量等措施,控制农业面源污染。

二是构建生态文明建设长效机制。坚持“多规合一”一张蓝图干到底,加强规划审批和后续监管,严守生态保护红线、环境质量底线、资源利用上线,引领生态环保、产业布局和项目建设。围绕海洋、水体、森林、大气等重点,加密和优化监测布点,提升监测信息化、自动化水平,统筹利用遥感监测、设置监测站和取样点等方式,逐步建立一套科学完整的环境监测评价体系。积极推广绿色能源,举办好第二届三亚国际能源论坛,加快建设天然气加气站、新能源汽车充电桩等配套设施,尽快推动公交车、出租车、公务用车、景区用车实现新能源汽车全覆盖。大力创建公交都市,倡导低碳出行。加快建设低碳智慧能源综合利用海棠湾示范区,扩大亚龙湾区域集中供能、供冷范围。积极推行城市新建建筑光伏发电技术应用,打造光伏发电示范小镇和示范村。大力发展装配式建筑,促进建筑业转型升级。加快编制海绵城市试点区域控制性详细规划,完成试点区域内道路、公园、广场、学校、小区和景区海绵化改造。加快推进三亚河国家湿地公园建设,加强三亚珊瑚礁国家级自然保护区等7个自然保护区的管理。尽快建立形式多样、绩效导向的生态补偿机制。加强环境保护立法工作,严格执法,提高全社会环保和守法意识。

(五)坚定不移深化改革开放,不断增强发展活力和动力。紧扣重点领域和关键环节,加快体制机制创新,扩大对外开放,提升三亚国际化水平,当好全省改革开放的排头兵。

一是持续深化改革。加快推进“多规合一”审批平台、电子证照库等信息化系统建设,理顺规划管理机制。深化“放管服”改革,继续精简行政审批服务事项,实现80%的政府服务事项“不见面审批”,积极推行省级产业园区“极简审批”。继续深化商事制度改革,全面推进企业登记全程电子化和电子营业执照广泛应用,完善“多证合一”,强化线上线下一体化监管,开展跨部门“双随机”联合抽查,完善跨部门信息共享和联合惩戒机制。大力推进强区扩权,做好新一轮市级权限下放和各区承接工作,完善市与区财政分税制管理,有效激发区级活力;继续探索区直管村(居)体制,推动“多村(居)合一中心社区”改革。全面完成国有独资企业的公司制改革,建立健全现代企业制度,提升国资监管和运营水平。通过委托管理、资产重组等方式做强国有企业。发展混合所有制经济,推动旅游板块国有企业整合上市。完成南新农场集团公司组建工作。支持和引导非公有制经济发展,坚决制止影响市场公平竞争的不合理行为,完善中小微企业贷款和信用担保服务,激发非公经济活力。

二是扩大对外开放。对标《三亚市国际化城市指标体系和评估体系》,加快建设符合国际标准的旅游信息咨询、旅游标识、游客服务中心、通信服务网点和外币兑换点等设施,加快制定与国际通行规则相衔接的旅游服务标准,今年围绕海鲜餐饮、热带水果等重点出台8项旅游地方标准。加强旅游警察、交通警察、涉旅一线服务人员和机关年轻干部的外语能力培训。充分利用博鳌亚洲论坛年会等平台,做好城市推介,积极引进和举办国际品牌赛事与重大活动。巩固和加密现有国际航线,积极拓展新开航线,加快提升航空保障能力和国际通达性,努力将三亚建设成为国际航空枢纽,今年争取新开通8~10条国际航线。成立三亚国际旅游营销中心,结合航线开辟情况,加强与知名国际旅游商、旅游类主流媒体的营销合作,创新城市品牌形象宣传和旅游精准促销,争取今年入境游客增长35%左右。加强与国际邮轮公司的合作,开辟更多邮轮航线,争取设立“中国邮轮旅游发展实验区”。聚焦特色优势农产品和服务贸易,培育壮大外贸企业,推动跨境电商发展。创造条件打造海上丝绸之路服务基地和泛南海国际贸易节点。完善总部经济发展和人才引进等政策,吸引更多企业到三亚设立总部、区域总部或独立核算的分支机构,吸引国际化人才进驻。大力开展友城交往,依托南海佛学院等平台,加强教育、文化、艺术等领域的对外交流合作。

(六)坚持以人民为中心,在发展中保障和改善民生。加快实施幸福民生行动计划,落实10个方面69项为民办实事事项,努力补齐民生短板,满足人民日益增长的美好生活需要。

一是优先发展教育事业。今年要新建、改扩建13所幼儿园、6所小学、5所中学,新增公办幼儿园学位2610个、小学学位2000个、中学学位1500个。深化与教育先进地区和机构的合作交流,充分发挥人大附中三亚学校、上海外国语大学三亚附中等学校的带动作用,做好西南大学托管市第六中学、海南中学托管市实验中学工作。坚持“一校一策”,提升教育质量。做实用好教育基金会,助推教育事业发展。大力实施“好校长、好教师”培养工程,面向全国招聘优秀校长、骨干教师,实施中小学校长、教师全员培训和考试测评,建立完善奖惩机制。切实保障教师待遇,特别是要逐步提高山区教师待遇。继续开展城乡教育对口帮扶,选派优秀教师到农村支教。加快荔枝沟教育产业园规划建设,大力发展职业教育,充分利用海南热带海洋学院、三亚学院等高校资源,鼓励特色学科建设发展,扩大教育对外开放。

二是提高医疗卫生服务水平。确保市妇幼保健院新院区建成投用,抓好市疾病预防控制中心、市传染病医院建设,加快推进市人民医院新院区、市中医院扩建项目。开展好市人民医院与上海交大医学院附属仁济医院、市妇幼保健院与上海交大医学院附属上海儿童医学中心合作共建工作,加强各医院重点特色学科建设。积极推进基层医疗卫生机构标准化建设,抓紧推动崖城卫生院迁址新建等项目,推动原镇区基层卫生院与农垦基层医院整合工作,推动社区健康服务中心(服务站)建设。加快组建区域性医疗服务联合体,稳步实施分级诊疗制度。完善计生服务管理,提高出生人口素质。进一步强化食品药品安全监管。

三是加快发展公共文化体育事业。加快建设区级图书馆、文化馆、全民健身中心、综合文体服务中心和行政村(社区)基层综合性文化服务中心,深入实施文化惠民工程,打造基层公共文体服务“15分钟文体圈”。加强崖州民歌、黎族打柴舞等非物质文化遗产的保护、利用和传承。挖掘利用好本土文化,推出更多体现三亚特色的文艺精品。积极创建“东亚文化之都”国家级公共文化服务示范区。促进全民健身和全民健康深度融合,创建国家运动健康城市。

四是织密扎牢民生保障网。落实创新创业扶持政策,加强职业技能培训,力争今年新增城镇就业岗位2万个,完成农村劳动力转移就业6000人,城镇登记失业率控制在4%以内。大力实施“全民参保登记计划”和建筑业“同舟计划”,进一步扩大社保覆盖面,打造“一站式”社保结算平台,完善社会救助体系。加快保障性住房、棚改安置区建设,抓好农村危房改造,妥善解决本地居民和“新市民”的基本住房需求,逐步建立多主体供给、多渠道保障、租购并举的住房体系。继续实施“菜篮子”工程,扩大平价蔬菜销售网点覆盖面,加强价格监管和调控,保持物价基本稳定,完善低收入群体价格补贴与物价上涨联动机制。

三、加强政府自身建设

坚持以习近平新时代中国特色社会主义思想为指导,切实增强“八种本领”,忠诚履职,务实担当,努力提升政府服务效能和治理现代化水平,真正做到为人民服务,让人民满意。

(一)加强政治建设。认真贯彻落实党的十九大精神,不忘初心,牢记使命,增强“四个意识”,坚定“四个自信”,严格遵守政治纪律和政治规矩,坚决维护以习近平同志为核心的党中央权威和集中统一领导,坚决贯彻落实中央方针政策以及省委省政府和市委的决策部署。坚持推进“两学一做”学习教育常态化制度化,认真开展“不忘初心、牢记使命”主题教育。

(二)坚持依法行政。完善政府权力清单、责任清单和负面清单,健全依法决策机制,增强公共政策制定的透明度和公众参与度,做好政务公开工作。加强公共资金、资产、资源的监督管理。坚持问题导向,加强和改进政府立法工作。加大普法力度,加快市、区、基层公共法律服务平台建设。改进综合执法体制,完善执法程序,明确操作流程,做到严格执法、规范执法、文明执法。坚持向市人大报告工作、向市政协通报工作制度,认真执行市人大及其常委会的决议、决定,自觉接受人大法律监督和政协民主监督,认真办理人大代表建议和政协提案。加强同各民主党派、人民团体和社会各界人士的联系。

(三)提升行政效能。加强政府执行力建设,坚持马上就办、办就办好,完善工作责任制,强化督查问责。落实容错纠错等机制,鼓励政府工作人员敢于担当、创新实干。结合“智慧城市”建设,加快政务信息系统互联互通,打通信息壁垒,加强政务信息整合共享,提高行政决策水平和管理效率。在推行“不见面审批”的基础上,构建多层次、多维度的政务服务应用体系,并以打造智能村居为抓手,进一步加强基层便民服务站建设,让群众少跑腿、好办事。

(四)改进工作作风。严格落实中央八项规定实施细则精神和省、市实施细则,驰而不息纠正“四风”。大力整治文山会海,改进文风会风。坚持把群众路线贯穿政府工作全过程,深入基层,了解实情,多办实事。结合监察体制改革试点工作,加强政府廉政建设,推进廉政风险防控工作,深入开展审计监督,强化重点领域和关键岗位监督,坚决查处不正之风和腐败行为。正确处理政府与企业的关系,加快构建亲清新型政商关系。

各位代表,新时代开启新征程,新使命呼唤新担当。让我们更加紧密地团结在以习近平同志为核心的党中央周围,以习近平新时代中国特色社会主义思想为指导,在省委、省政府和市委的坚强领导下,不忘初心,牢记使命,砥砺前行,加快建设世界级滨海旅游城市,为当好建设美好新海南的排头兵而努力奋斗!
